# Polyparaneoplastic Manifestations of Malignant Thymoma: A Unique Case of Myasthenia, Autoimmune Hepatitis, Pure Red Cell Aplasia, and Keratoconjunctivitis Sicca

**DOI:** 10.7759/cureus.1374

**Published:** 2017-06-20

**Authors:** Doron Feinsilber, Katrina A Mears, Brian L Pettiford

**Affiliations:** 1 Hematology/Oncology, Medical College of Wisconsin/Froedert Cancer Center; 2 Department of Ophthalmology, Retina Consultants of Southwest Florida, National Ophthalmic Research Institute, Fort Myers, Fl; 3 Department of Surgery, Ochsner Clinic Foundation

**Keywords:** thymoma, myasthenia, keratoconjuncivitis sicca, autoimmune hepatitis, plasmapharesis, pure red cell aplasia

## Abstract

Thymomas are relatively uncommon malignancies of the anterior mediastinum and present with four distinct histological types based on the specific epithelial to lymphocyte ratio: spindle cell, epithelial predominant, lymphocyte predominant, or mixed. Each histologic type of thymoma has a propensity for local invasion and metastasis and can have a wide variety of paraneoplastic manifestations, myasthenia being the most common. We present a unique case of a 34-year-old African-American female who initially presented with a history of profound weakness with repetitive motion, shortness of breath, horizontal nystagmus, persistent anemia, keratoconjunctivitis sicca, and what was initially thought to be azithromycin-induced hepatitis. Upon left anterior thoracotomy with biopsy of the mediastinal mass, pathology yielded a lymphocyte-predominant (B1), Masaoka stage IVA invasive thymoma with pericardial extension. This case illustrates the clinical significance of considering a multitude of extrathymic paraneoplastic manifestations, each with a unique physiological mechanism.

## Introduction

Thymomas are neoplasms that develop from the epithelium of the thymus gland with a mixture of lymphocytes and epithelial cells in varying proportion. Thymic epithelial tumors are classified as spindle, lymphocytic, mixed, epithelial, and as thymic carcinoma [1]. Radiographic assessment of invasion can be difficult and may not be evident until surgical exploration. Invasive thymomas and thymic carcinomas are rare and represent approximately 0.2% to 1.5% of all malignancies. The overall incidence of thymoma is 0.15 cases per 100,000 [2]. Thymomas are usually considered indolent tumors that tend to recur locally, whereas thymic carcinomas are characterized by invasion and regional spread including drop metastases which are defined as intradural extramedullary spinal metastases that arise from intracranial lesions. Thymic carcinomas also carry a much greater risk of relapse and death [3]. Paraneoplastic disorders most commonly seen with thymomas include myasthenia, pericarditis, Addisons disease, autoimmune hepatitis, neutropenia, alopecia areata, Cushing's syndrome, hemolytic anemia, nephrotic syndrome, limb encephalopathy, pure red cell aplasia, polymyositis, stiff man syndrome, aplastic anemia, and systemic lupus erythematous. We present a case of an invasive thymoma in a young African-American female who presented with a plurality of unique paraneoplastic manifestations.

## Case presentation

A 34-year-old African-American female with a medical history of obesity, depression, and autoimmune hepatitis initially thought to have been drug-induced from azithromycin presented with dry eyes, fatigue, occasional blurred vision, and progressive shortness of breath. Keratoconjunctivitis was diagnosed by her ophthalmologist who prescribed cyclosporine ocular drops.

### Autoimmune hepatitis

In June 2010, the patient’s gynecologist prescribed a 3-day course of azithromycin after a cervical biopsy. The patient subsequently developed pruritus that worsened during the following month, and her liver function test (LFT) values were elevated: alanine transaminase 325 U/L, aspartate transaminase 682 U/L, alkaline phosphatase 174 U/L, and total bilirubin 14.2 mg/dL. Laboratory tests were negative for viral hepatitis, and alpha-fetoprotein levels were within normal limits. Liver biopsy showed cholestasis and large duct obstruction. The patient received ursodeoxycholic acid and steroids without any improvement in symptomatology or chemistries. A repeat liver biopsy was performed eight months later when her alkaline phosphatase level increased to 1165 U/L and total bilirubin increased to 20.4 mg/dL, demonstrating acute cholangitis and biliary cirrhosis. Etiology of liver failure at that time was determined to be secondary to azithromycin. The patient was placed on a liver transplant evaluation list, but 14 months after exposure, the patient’s LFTs normalized. Despite LFT normalization, the patient had repeat office visits with complaints of pruritus. After dark marks appeared on her extremities, the patient was referred to dermatology, and she was diagnosed with lichen simplex chronicus.

### Pure red cell aplasia

Approximately five years after her initial presentation in 2010 the patient became progressively anemic with a hemoglobin nadir of 7.5 g/dL and a mean corpuscular volume nadir of 77 fL, whereas her usual baseline hemoglobin was 12.0 g/dL. The patient was noted to be markedly iron deficient with a ferritin nadir of 8 ng/mL and a saturated iron nadir of 2%. All other cell lines were in the normal range. The patient was referred to the hematology clinic and was treated for profound iron deficiency anemia. She required multiple iron gluconate infusions but remained persistently anemic despite receiving monthly iron infusions.

### Myasthenia

**Figure 1:**
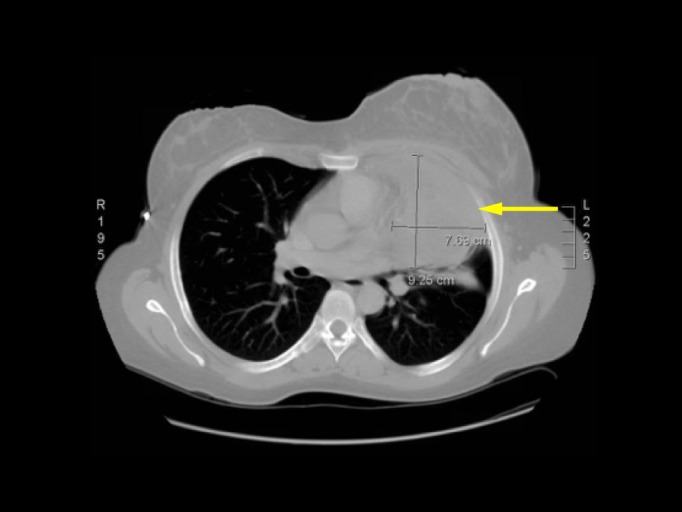
Computed tomography (CT) of the chest. A CT of the chest with contrast revealed a 7.69 cm x 9.25 cm anterior mediastinal mass with central calcification and extension into the anterior left chest abutting the pleura (yellow arrow).

The patient developed myasthenic symptoms, including increased fatigue with repetitive motion, diplopia, blurred vision, productive cough, and horizontal nystagmus. Computed tomography (CT) of the chest with contrast revealed a 6.5 × 9 cm anterior mediastinal mass with central calcification and extension into the anterior left chest abutting the pleura (Figure 1).

An additional pleural-based soft tissue density measuring 4.6 × 1.8 cm was seen along the inferior posterior aspect of the left hemithorax.

**Figure 2:**
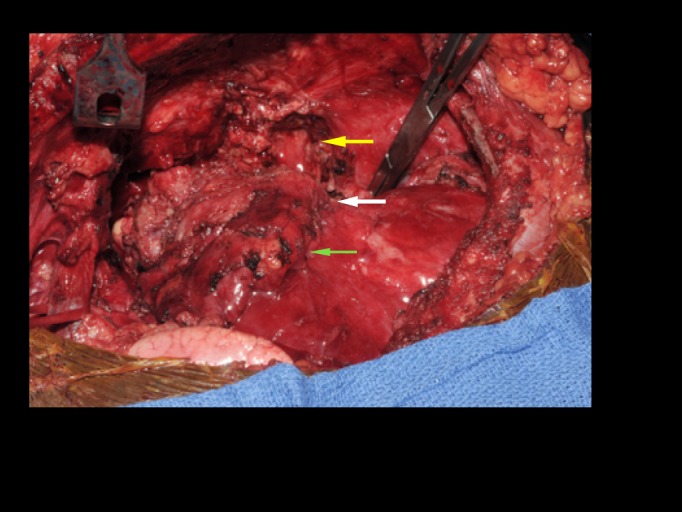
Intraoperative findings. Intraoperative findings demonstrated involvement of the pericardium (green arrow) and phrenic nerve (white arrow) as well as invasion into the adjacent left upper lobe (yellow arrow).

The patient underwent a left anterior thoracotomy with biopsy of the mediastinal mass. Intraoperative findings demonstrated involvement of the pericardium and phrenic nerve, as well as invasion into the adjacent left upper lobe (Figure 2).

**Figure 3:**
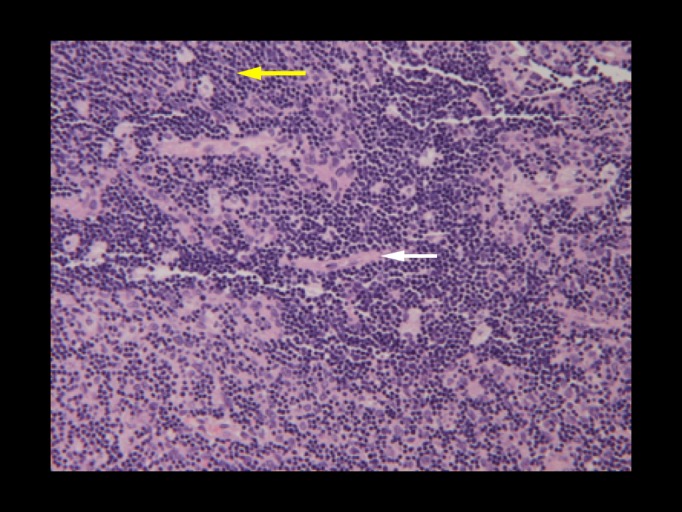
Surgical histopathology of thymic mass. Sections from the tumor showed a lymphocyte-predominant population with scattered starry sky-appearing cells representing macrophages (yellow arrow). Admixed with these lymphocytes were cells with vesicular chromatin and prominent nucleoli representing epithelial cells. Dense fibrous tissue traversed throughout the tumor and was extensively invaded by the tumor cells (white arrow).

Frozen section analysis suggested lymphoma; however, the final pathologic analysis revealed an invasive thymoma, lymphocyte predominant, type B1. Sections from the tumor showed a lymphocyte-predominant population with scattered starry sky-appearing cells representing macrophages. Admixed in these lymphocytes were cells with vesicular chromatin and prominent nucleoli representing epithelial cells. Dense fibrous tissue traversed throughout the tumor and was extensively invaded by the tumor cells. The tumor also invaded the fibroadipose tissue. Scattered rare Hassall’s corpuscles were also seen (Figure 3).

Immunostains CD3, CD5, and AE1/3 were positive, with few cells showing CD20 positivity. EMA, S-100, and CD30 were negative. CD15 staining showed nonspecific positivity.

Echocardiogram performed four days postoperatively revealed a mildly depressed left ventricular ejection fraction of 45-50% due to the extent of pericardial involvement. A small pericardial effusion was noted along the right ventricular wall. However, no evidence of electrical alternans was seen on EKG. Neoadjuvant chemotherapy was recommended, and the patient received two cycles of dose-reduced chemotherapy secondary to the cardiomyopathy: cisplatin 50 mg/m^2^, doxorubicin 50 mg/m^2^, cyclophosphamide 500 mg/m^2^ (CAP). Left ventricular ejection fraction was reassessed two months later and showed mild improvement at 50-55%.

After the patient completed two rounds of neoadjuvant chemotherapy, repeat CT scan revealed that the size of the mass had decreased. Following her third cycle of chemotherapy, the patient presented with increasing weakness and shortness of breath. Repeat CT scan revealed the progression of the disease. The patient was reassessed for thymoma resection. On the morning of surgery, she underwent plasmapheresis. After thoracosternotomy, the tumor was found infiltrating the pericardium and phrenic nerve and encasing multiple left upper lobe pulmonary arterial branches. The entire mass, including the involved pericardium, phrenic pedicle, and a portion of the left upper lobe, were resected en bloc (Figure 2). Pathology revealed invasive thymoma, World Health Organization (WHO) classification type B3, with minor B2 component.

The patient’s postoperative course was complicated only by a localized wound infection that resolved with antibiotic therapy drainage and packing. The patient was referred to radiation oncology and was scheduled to receive 54 grays to the tumor bed. The patient still has visual changes and red cell aplasia but has reported improvement in her respiratory symptoms and strength.

## Discussion

### Mechanism for autoimmune hepatitis

Paraneoplasia is multifaceted and requires a unique lattice of both humoral and cell-mediated responses. Thymoma-induced autoimmune hepatitis has several proposed mechanisms of pathogenesis, namely autoantigen presentation in the context of MHC II of UGT1A6 and production of UGT1A6 antibodies [4]. Other proposed mechanisms seen in mouse models are gene mutations for TRAF6 that cause depletion of medullary thymic epithelial cells that in turn specifically causes a hepatic autoimmune response [4-6].

### Mechanism for myasthenia

Our case illustrates that thymomas can present with paraneoplastic manifestations. A proposed mechanism of these paraneoplasm, especially myasthenia, is their autoimmunity to anti-acetylcholine receptors (AChR), striated muscle proteins, and neuronal antigens [5]. It has been experimentally derived that a process of abnormal intratumoral positive selection occurs with upregulation of abnormal expression of cross-reacting epitopes with homology to AChR [5]. This immune dysregulation specifically has been described within category 1 malignant thymomas that have exposure prior to tumor resection. The tumor is covering the pulmonary artery trunk, ascending aorta, innominate artery, and pericardium. There is also adhesional formation on the left anterior chest wall with intratumoral T cells with the immature phenotype CD1^+^/CD4^+^/CD8^+^/CD3^-^. It is proposed that the development of immature thymocytes into autoaggressive mature T cells is present. Based on murine models, this process is mediated by epithelium-mediated negative selection [5].

### Mechanism for pure red cell aplasia

At the age of 34 years, our patient was relatively young. Most published cases of invasive thymomas occur around the fifth decade of life when thymic T cells can potentially undergo gene rearrangements and have difficulty managing suppressor T cells and antigen anergy [6].

### Prognosis

The degree of invasiveness and completeness of resection are the best predictors of relapse-free survival. The Masaoka Staging System is used widely for grading, staging, and for treatment planning [1]. For our patient, the extracapsular extension into the pectoralis minor muscle and extension into the left upper lobe suggested the potential for a high locoregional recurrence rate with surgery alone [8]. Accordingly, adjuvant external beam radiation was indicated [10].

### Case-specific management

National Comprehensive Cancer Network guidelines indicate that patients with stage III or IV disease should receive adjuvant chemotherapy and radiation therapy [2]. One unique feature of our case is that the patient had significant pericardial involvement that required a dose-reduced regimen of an anthracycline. Despite two cycles of doxorubicin, cyclophosphamide, and cisplatin, the patient’s scans showed progressive disease and she also had worsening symptoms; therefore, we felt surgical intervention was necessary, and waiting for concurrent radiation therapy would have resulted in an unacceptable surgical treatment delay. Additionally, elevation in total bilirubin was approaching 2.0 mg/dl which would have further limited our ability to utilize anthracyclines [7-9].

Plasmapheresis prior to this patient’s surgery was necessary to remove anti-AChR antibodies from circulation to minimize anesthetic complications and prolonged intubations. Depolarizing neuromuscular blockade anesthetic agents cause unpredictable respiratory failure and post extubation in myasthenia gravis patients, as the available amount of AChR is not consistent because of the presence of the anti-AChR antibodies [10].

Post-thoracosternotomy biopsy pathology revealed a different stage than prethoracotomy biopsy pathology. The WHO classification of the thymoma changed from B1 to B3 with a minor component of B2. This change was likely attributable to a treatment effect, as neoadjuvant therapy could potentially have depleted the lymphocytes.

## Conclusions

Thymomas with distinct multiparaneoplastic features mandate a multidisciplinary approach. Early identification and treatment are needed to ensure an overall positive outcome. A detailed history and physical examination can identify paraneoplastic processes in patients with thymoma. Specific attention should be paid to perioperative medical management, including preoperative plasmapheresis and postoperative intravenous immunoglobulin when indicated. The goal of surgery is complete resection including adjacent involved structures. Adjuvant therapy such as external beam radiation is indicated in cases of transcapsular invasion. The collaborative efforts of medical oncology, thoracic surgery, pulmonary, ophthalmology, and radiation oncology ultimately enhanced the clinical outcome.
